# Adaptive hyperparameter updating for training restricted Boltzmann machines on quantum annealers

**DOI:** 10.1038/s41598-021-82197-1

**Published:** 2021-02-01

**Authors:** Guanglei Xu, William S. Oates

**Affiliations:** 1 Department of Mechanical Engineering, College of Engineering, Florida A&M-Florida State University, Tallahassee, FL 32310 USA; 2 Florida Center for Advanced Aero-Propulsion (FCAAP), Tallahassee, FL 32310 USA

**Keywords:** Computational science, Statistics, Mechanical engineering

## Abstract

Restricted Boltzmann Machines (RBMs) have been proposed for developing neural networks for a variety of unsupervised machine learning applications such as image recognition, drug discovery, and materials design. The Boltzmann probability distribution is used as a model to identify network parameters by optimizing the likelihood of predicting an output given hidden states trained on available data. Training such networks often requires sampling over a large probability space that must be approximated during gradient based optimization. Quantum annealing has been proposed as a means to search this space more efficiently which has been experimentally investigated on D-Wave hardware. D-Wave implementation requires selection of an effective inverse temperature or hyperparameter ($\beta$) within the Boltzmann distribution which can strongly influence optimization. Here, we show how this parameter can be estimated as a hyperparameter applied to D-Wave hardware during neural network training by maximizing the likelihood or minimizing the Shannon entropy. We find both methods improve training RBMs based upon D-Wave hardware experimental validation on an image recognition problem. Neural network image reconstruction errors are evaluated using Bayesian uncertainty analysis which illustrate more than an order magnitude lower image reconstruction error using the maximum likelihood over manually optimizing the hyperparameter. The maximum likelihood method is also shown to out-perform minimizing the Shannon entropy for image reconstruction.

## Introduction

**Figure 1:**
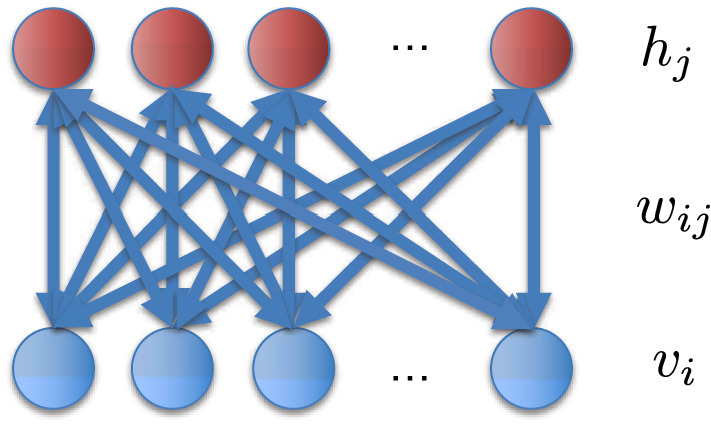
Illustration of a Restricted Boltzmann Machine (RBM) bipartite graph where $v_i$ are visible nodes, $h_j$ are hidden nodes and $w_{ij}$ are the weights connecting the hidden and visible nodes.

Restricted Boltzmann Machines (RBMs) are commonly used as a fundamental building block for deep neural networks in machine learning algorithms^1^. An RBM consists of a bipartite graph that contains two layers of nodes that are fully connected with zero inter-layer connections; see Fig. 1. This graph structure contains unknown parameters ($w_{ij}$) that act as the network edge weights between nodes. The additional nodal source parameters are called the biases ($b_i, c_j$). An optimal set of weights and hidden layer biases are found through optimization of a Boltzmann distribution of energies given a set of input data applied to the visible layer of the RBM. Training such networks, via optimization and sampling, requires large computational resources as the size of the network grows^2^.

Quantum annealing algorithms have been proposed to efficiently search over the large parameter space to accelerate the RBM machine learning training process^3^. The RBM assumes data follows a Boltzmann distribution which contains a normalization term, i.e., the partition function. During gradient based optimization, this term requires sampling over a large configuration space thus creating a bottleneck in the optimization process. Approximations are typically introduced by sampling over a subspace. One popular method is contrastive divergence where only one Markov step is taken per optimization step (i.e., an epoch)^4^. This algorithm, referred to as CD-1 can be modified to take *n* steps (CD-n) and thus minimizes the effect of global optimization errors that may occur using CD-1 with the trade-off of more computational requirements. These methods typically treat the inverse temperature $\beta$ in the Boltzmann distribution fixed to a value of 1. In contrast, recent research^5^ has incorporated variable temperature into RBMs to enhance training and predictions from the neural network. Quantum annealing hardware, based on quantum adiabatic optimization^6^, has been proposed to help address this limitation by searching over a broader number of configurations using quantum algorithms to minimize uncertainty in finding the global optimal solution. As quantum hardware grows in qubit size and connectivity, larger RBMs may be implemented to handle larger amounts of information to provide more robust predictions of complex systems which, in principle, cannot be done on classic computers.

**Figure 2:**
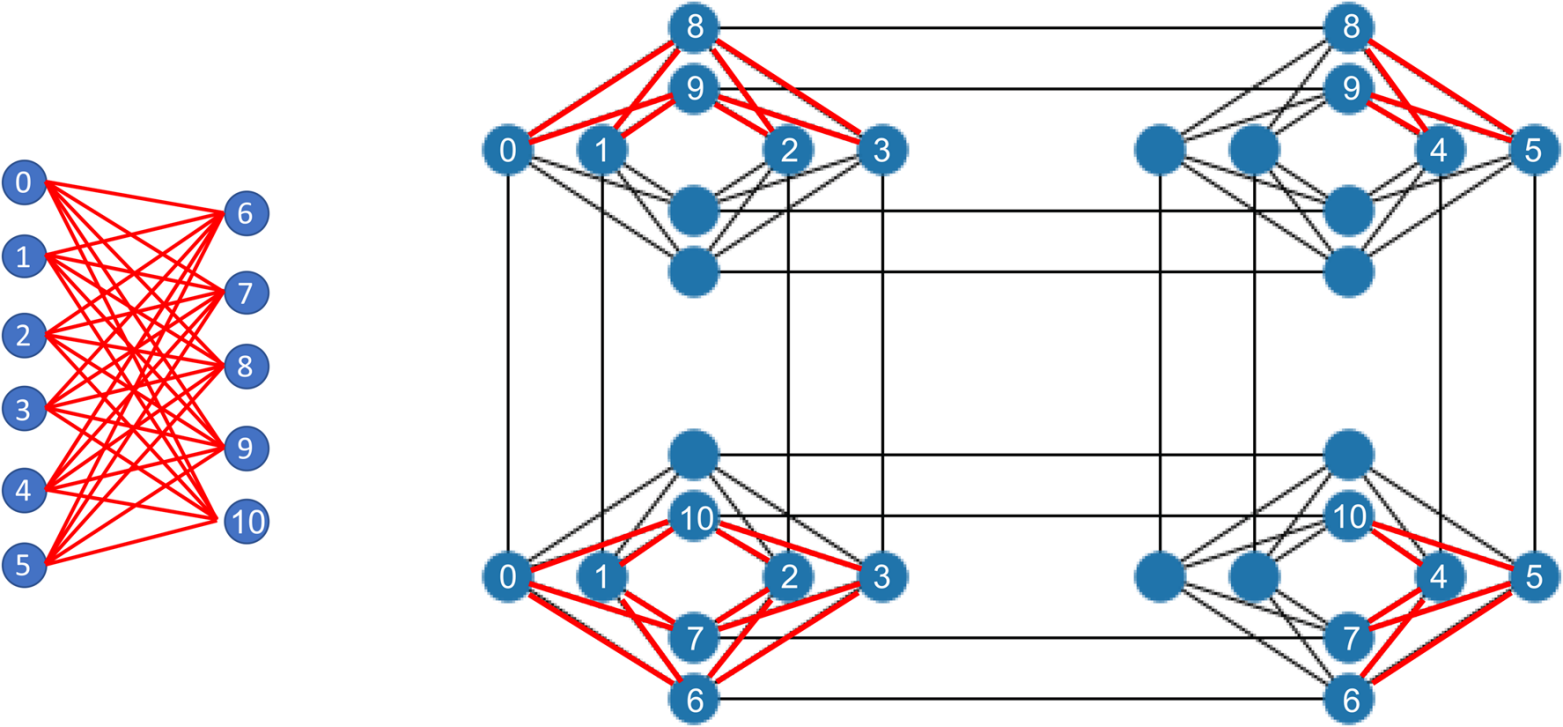
Illustration of a Restricted Boltzmann Machine (RBM) bipartite graph of size $6 \times 5$ (left), and embedded Chimera graph^7^ on D-Wave hardware (right).

An optimization methodology has been proposed that takes advantage of adiabatic state transformation while carefully controlling the annealing schedule^8^. Annealing on the D-Wave starts with a ‘transverse-field’ Hamiltonian ($$H_d = -\sum _i^N \sigma _i^x$$) of transverse spins $$\sigma _i^x$$ over *N* qubits. The target Hamiltonian is $$H_p = \sum _{i,j} J_{ij}\sigma _i^z \sigma _j^z + \sum _i h_i \sigma _i^z$$ where $$J_{ij}$$ and $$h_i$$ are controllable parameters that are mapped to the RBM weights ($$w_{ij})$$ and biases ($$b_i, c_i$$). Annealing starts by initiating the hardware to the ground state in $$H_d$$ and then the system evolves to $$H_p$$ over a predefined schedule. This is done by starting in a superposition of $$\sigma _i^x$$ and ideally ending in the ground state of $$H_p$$ where the unknown $$\sigma _i^z$$ spin states are measured given some fixed $$J_{ij}$$ and $$h_i$$ values. This annealing schedule follows $$H(s) = A(s)H_d + B(s)H_p$$ where $$A(0)>>B(0)$$ and $$B(1)>>A(1)$$ where *s* is some normalized time that is defined to be less than the decoherence time. It has been suggested that pausing *s* at intermediate times between 0 and 1 increases the probability of reaching a global minimum^9^. However, the pausing time depends on the spectrum of energy values of *H*(*s*) which are unknown for the machine learning problem because the biases and weights change during training. Given this uncertainty, we evaluate a broad range of pausing times to determine if any correlation exist between our hyperparameter updating schemes and pausing times during RBM training.

Several methods have been considered to optimize $\beta$ for generalizing machine learning training on D-Wave hardware^10–12^. One simple method is to determine a fixed optimal value before the training^10^. This method requires comparing the probability distributions between randomly generated graphs ($n+m$ nodes), which are calculated from all possible configurations ($2^{(n+m)}$), and feedback from errors on D-Wave hardware. This approach is limited to specific training tasks and results in significant computational difficulties when considering large systems. The estimation of the effective temperature requires additional calculations from samples measured from D-Wave hardware. By regrouping all the *R* samples into $K = \lceil \sqrt{R} \rceil$ bins, the *K* averaged energies are used to obtain ${\mathcal {O}}(K^2)$ data points (energy pairs) from linear regression to estimate the effective temperature. A large amount of post processing is required during each iteration which can require considerable computational resources. One recent article also performed effective temperature estimation by minimizing the difference between the empirical distribution and the exact Boltzmann distribution^12^. However, the method can only be done for sufficiently small problems.

Here we treat the unknown $\beta$ as a hyperparameter that aims to accommodate measurement noise, hardware imperfections, embedding errors used to map RBM graphs onto quantum hardware, and the working temperature of the hardware. This hyperparameter is inferred by either: (1) maximizing the likelihood or (2) minimizing Shannon’s entropy. These two methods treat $\beta$ as an unknown hyperparameter that is updated at each classical step of the RBM optimization (i.e., epoch) simultaneously along with the weights and biases. It is important to note that this $\beta$ is only applied onto D-Wave hardware which indirectly influences updates to the weights and biases. The sigmoidal function and direct optimization of the weights and biases neglect the effect of the inverse temperature essentially setting it to one. We find that these two methods outperform manually controlling $\beta$ on D-Wave hardware as done elsewhere^10^. All the results shown in our analyses were validated using $7\times 7$ pixelated bars and stripes (BAS) random images. This size was based on a balance of the largest pixel density and a set of hidden nodes that could be embedded within the D-Wave chimera graph structure. It included 49 visible nodes and 25 hidden nodes. These 74 RBM nodes were embedded onto the D-Wave chimera graph using 668 qubits. An example of a RBM that requires hardware embedding is shown in Fig. 2. When manually controlling $\beta$, we consider fixed $\beta$ values that give the lowest image reconstruction error again noting that this $\beta$ is only applied on the D-Wave hardware but not within the sigmoidal functions of the RBM. We then linearly ramp $\beta$ from a low value to the value giving the lowest reconstruction error for comparisons to maximum likelihood and minimum Shannon entropy updating. For the maximum likelihood or minimum Shannon’s entropy approach, $\beta$ is updated after each epoch using information about the magnitude of energy output from D-Wave hardware. For the case of maximum likelihood, $\beta$ is updated based on the differences in the average energy while minimum Shannon entropy updates $\beta$ by reducing the variance of the D-Wave energy. It is shown that maximizing the likelihood is about 23 times better than the linearly ramping method for any pausing time. Similarly, this method is also more than 15 times better than minimizing Shannon’s entropy. In all cases, $\beta$ increases per epoch; however, the rate at which it increases significantly differs for each method.

In the following sections we first discuss details describing the optimization methodology and experimental implementation on D-Wave hardware. We then evaluate the results using Bayesian uncertainty analysis to quantify differences in reconstruction error for the different hyperparameter updating methods. This is followed concluding remarks and detailed derivations important for hardware implementation in the “Methods” section.

## Results

### Restricted Boltzmann Machines

A brief description of the theory and application of RBMs for machine learning is described in this section to support hyperparameter optimization. Core to its formulation is the energy associated with the nodes and their interactions. The energy of this network is described by

1 $$\begin{aligned} E = - \sum _i b_i v_i - \sum _j c_j h_j - \sum _{i,j} w_{ij} v_i h_j \end{aligned}$$

where all variables have been previously described in the introduction and Fig. 1.

The RBM model assumes the probability of configurations with a specific energy value ($E_k$) follows the Boltzmann distribution ${\displaystyle P_k = \frac{e^{-E_k}}{Z}}$ where the distribution is normalized by the partition function ${\displaystyle Z = \sum _k e^{-E_k}}$ and *k* denotes the discrete number of different configurations of the nodal values of $v_i$ and $h_i$.

The training procedure requires updating the biases and weights ($\theta = [b_i, c_i, w_{ij}]$) so that the visible nodes can be reproduced through the conventional reconstruction process. This process entails inputs from the visible to the hidden nodes which is then reconstructed as $v_i \rightarrow h_i \rightarrow v_i^{rec}$ where each $\rightarrow$ operation represents calculations done by the sigmoid function^13^. In practical training, initial values of biases and weights can be set arbitrarily. In this article, the biases are set to be initially zero and the weights are initially proportional to a normal distribution with the small prefactor value of 0.01.

Instead of directly maximizing the Boltzmann distribution, we minimize its negative log-likelihood (${\displaystyle {\mathcal {L}}(\theta ) = -\sum _k \log P_k}$) using the gradient descent algorithm assuming a continuous distribution. This is done by taking variations of ${\mathcal {L}}(\theta )$ with respect to $\theta$ according to

2 $$\begin{aligned} \delta \mathcal {L(\theta )} = -\sum _k \delta \left( \log P_k \right) = -\sum _k \left[ \sum _\theta \frac{\partial }{\partial \theta } \left( \log P_k \right) \right] \cdot \delta \theta = -\sum _\theta \left[ \sum _k \frac{\partial }{\partial \theta } \left( \log P_k \right) \right] \cdot \delta \theta . \end{aligned}$$

Additional details on this method can be found elsewhere^3^.

A general form of the updating scheme can be derive from the variation of ${\mathcal {L}}(\theta )$ as

3 $$\begin{aligned} \frac{\partial ( - \log P_k ) }{ \partial \theta }&= \frac{\partial E_k }{ \partial \theta } - \big \langle \frac{\partial E }{ \partial \theta } \big \rangle \end{aligned}$$

where the $\langle \cdot \rangle$ represents the ensemble average value of the Boltzmann distribution. The first term is called the positive phase and the second term is called the negative phase. The parameters $\theta$ may be updated according to

4 $$\begin{aligned} \delta \theta&= -\eta \left[ {\frac{\partial {\overline{E}} }{ \partial \theta }} - \big \langle \frac{\partial E }{ \partial \theta } \big \rangle \right] \end{aligned}$$

where $\eta >0$ is the learning rate and ${\overline{E}}$ denotes the averaged energy calculation from the training data. From (4), the biases and weights are explicitly

5 $$\begin{aligned} \delta b_i&= - \eta [ -\overline{v_i} - \langle -v_i \rangle ] = \eta [\overline{v_i} - \langle v_i \rangle ] \end{aligned}$$

6 $$\begin{aligned} \delta c_j&= \eta [\overline{h_j} - \langle h_j \rangle ] \end{aligned}$$

7 $$\begin{aligned} \delta w_{ij}&= \eta [\overline{v_i h_j} - \langle v_i h_j \rangle ]. \end{aligned}$$

These ensemble averages often require large amount of computing power to reach high accuracy for large sets of data (e.g., CD-n). On classical computers, contrastive divergence (CD) is often implemented to estimate the ensemble average value. In comparison, the D-Wave quantum annealer is believed to follow a Boltzmann distribution^3^, which can provide an alternative approach to calculate the ensemble average more efficiently.

### Implementation on D-Wave hardware

Here we describe how the RBM energy is applied to D-Wave hardware for this class of machine learning optimization problems. It is within this part of the algorithm that $\beta$ is introduced to accommodate statistical distributions on hardware. A class of optimization problems, known as quadratic unconstrained binary optimization (QUBO), are described by a cost function

8 $$\begin{aligned} E_{DW} = \sum Q_{ii} x_i + \sum Q_{ij} x_i x_j \end{aligned}$$

that can be applied to D-Wave architecture on its Chimera graph, where $x_i = \{0,1\}$ for all *i* qubits. It is possible to calculate an ensemble average by sampling the visible and hidden nodes on the RBM $\{v_i,h_i\}$ that corresponds to the D-Wave QUBO.

The QUBO is constructed from the transformation

9 $$\begin{aligned}&Q_{ii} = {\left\{ \begin{array}{ll} - b_i / \beta &{} \quad \text {if } i \le M \\ - c_{i-M} / \beta &{} \quad \text {if } M<i \le M+N \\ \end{array}\right. } \nonumber \\&Q_{ij} = - w_{ij} /\beta \qquad \text {if } i \ne j \end{aligned}$$

where the hyperparameter $\beta$ is introduced to construct a proper Boltzmann distribution such that $\beta E_{DW} = E$.

When the QUBO matrix $Q_{ij}$ is applied to the D-Wave quantum annealer, the output are vectors $\{v_i,h_i\}$ exhibit certain experimental energy spectra over $E_{DW}$. This energy spectra is assumed to follow the Boltzmann probability density function. We calculate the expectation values: $\langle v_i \rangle , \langle h_i \rangle , \langle v_i h_j \rangle$ using 1000 samples from the D-Wave machine. Each individual sample undertakes one annealing process. The annealing time is set to 6 $\mu$s with a pausing $t_{pause} = 2~\mu$s. We used the built-in minor-miner embedding algorithm^14^ to represent the RBM graph on the Chimera graph^7^. The D-Wave machine uses a major voting algorithm to determine the logical qubit values represented by each embedded chain. As previously discussed, RBM parameter updating is typically conducted by manually optimizing the hyperparameter $\beta$ to fixed values or linear ramping it over each epoch. Since $\beta$ can depend on many unknown factors^3,10^, we implement the adaptive schemes to update $\beta$ concurrently while updating the parameters within the RBM neural network.

### Hyperparameter updating

We present details on the three approaches used to vary $\beta$ during RBM training. The methodologies are characterized by 1) manual control of $\beta$ through linear ramping, 2) maximizing the likelihood of the Boltzmann distribution and 3) minimizing the Shannon entropy. In all three cases, $\beta$ is updated per epoch either manually (case 1) or automatically (cases 2 or 3) while updating the weights and biases within the neural network. Key relations are given here while additional details can be found in the “Methods” section.

In all cases, $\beta$ is increased over the epochs with the goal of reducing errors associated with regenerating the correct Bars and Stripes (BAS) images. This increase in $\beta$ is loosely analogous to decreasing an effective temperature such that the optimal or near optimal solution is frozen at the final epoch. We find that the rate and magnitude of this increase in $\beta$ is important for both faster training and reaching the lowest asymptotic error as seen later in Fig. 4. We also emphasize that $\beta$ is not directly involved in the reconstruction error nor is it used in the sigmoidal function within the RBM.

### Linearly hyperparameter ramping

**Figure 3:**
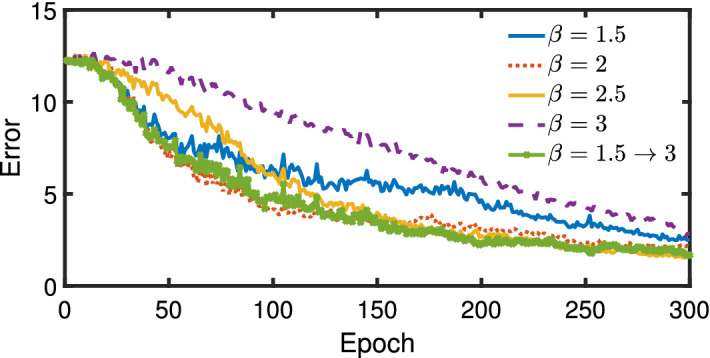
Plot of reconstruction errors during training with different values of fixed $\beta$ and linearly ramping $\beta$.

A series of test trials were conducted with constant values of $\beta$ to assess its effect on the steady-state error. As shown in Fig. 3, we find that different fixed values of $\beta$ cause different convergence rates and steady-state errors based on neural network training. Given this information, we linearly varied $\beta$ to improve the convergence rate and steady-state error for comparisons to the two adaptive schemes.

Figure 3 illustrates a trend of faster convergence to a steady-state error as $\beta$ decreases from 3 to 2 followed by a slower convergence of error when $\beta =1.5$. This set of data also shows a difference in the final error for an epoch of 300. The lowest error was achieved when $\beta =2.5$; however, it is unclear if the larger $\beta$ values have reached a steady state error at 300 epochs. This was not considered further as we focused on methodologies to achieve faster convergence without significantly sacrificing steady-state error.

Linear ramping $\beta$ from 1.5 to 3 describes training runs where $\beta$ is linearly increased over the epochs ($i_{epoch}$) according to $\beta (i_{epoch}) = 1.5 + 1.5i_{epoch} / 300$. By linearly increasing the hyperparameter, we find that the convergence rate is approximately equivalent to the best rate for a constant $\beta$ and it achieves practically the same steady-state error. Although linear ramping is an improvement over a constant value, it requires *a priori* knowledge of the range of values which may be problem dependent. Furthermore, a constant rate of increase of $\beta$ is unlikely optimal. Therefore, we present alternative methods to identify $\beta$ during the training process.

### Log-likelihood maximization

The quantum annealing process on the D-Wave machine results in a complex qubit state which creates challenges in identifying an *a priori* optimal value for $\beta$. This is further complicated over a set of training epochs where the weights and biases change their value to reduce the reconstruction errors. This motivates alternatives that update $\beta$ along with the weights and biases using a similar derivation as in (3). To illustrate this method, we again assume the D-Wave quantum annealing follows a Boltzmann distribution and define its energy distribution to be $E_{DW} = E/\beta$. We delineate the assumed Boltzmann distribution of the D-Wave annealer to be

10 $$\begin{aligned} P_{DW} = \frac{e^{-E_{DW}}}{Z_{DW}} = \frac{e^{-E/\beta }}{Z_{DW}} \qquad \text{ and } \qquad Z_{DW} = \sum e^{-E_{DW}} \end{aligned}$$

where *E* is as same as that given in (1).

The derivative of the negative log-likelihood requires

11 $$\begin{aligned} -\frac{\partial ( \log P_{DW} ) }{ \partial \beta }&= -\frac{1}{\beta ^2} ( E - \langle E \rangle ). \end{aligned}$$

**Figure 4:**
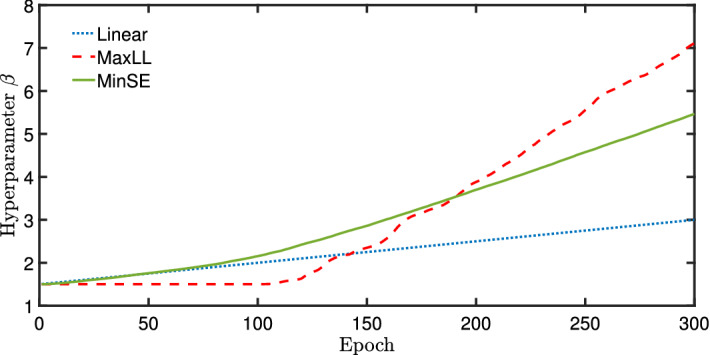
Plots of varying the hyperparameter $\beta$ using the three proposed schemes during training. The MaxLL and MinSE given here are representative of all 16 training cases for a fixed $s_{pause}$ value.

To minimize the negative log-likelihood with the same strategy shown in (2)-(4), the increment of $\beta$ averaged overall all configurations becomes

12 $$\begin{aligned} \delta \beta = - \eta \left( -\frac{1}{\beta ^2} \right) \left( {\overline{E}} - \langle E \rangle \right) = \frac{\eta }{\beta } \left( {\overline{E}}_{DW} - \langle E_{DW} \rangle \right) \end{aligned}$$

where ${\overline{E}} = \sum _{i=1}^{N} E$ represents the averaged value over all configurations. In the training process, the increment is set to $\delta \beta = \eta ( {\overline{E}}_{DW} - \langle E_{DW} \rangle )$ to have a similar form as the other parameters in (4). As shown in Fig. 4, $\delta \beta$ is relatively small in the beginning of training. Therefore, to compare this approach with the linear ramping cases, we neglect the dependence of $\beta$ within the relation $\delta \beta$. The learning rate $\eta$ was set to 0.1 which was the same as that used to update the weights and biases in (4)–(7).

### Shannon entropy minimization

In the third approach, $\beta$ is updated by minimizing Shannon’s entropy. This drives the system away from the high temperature state that corresponds to a system with a uniformly distributed configuration. Again using the gradient descent, we obtain an alternative scheme for updating $\beta$. We implement the approach by defining Shannon’s entropy with the Boltzmann probability distribution as

13 $$\begin{aligned} S&= - \sum _i P_i \log P_i \end{aligned}$$

14 $$\begin{aligned} P_i&= \frac{e^{-\beta E^i_{DW}}}{Z} \qquad \text{ and } \qquad Z = \sum _i e^{-\beta E^i_{DW}} \end{aligned}$$

The first derivative of the Shannon entropy with respect to $\beta$ is

15 $$\begin{aligned} \frac{\partial S}{\partial \beta } = \beta \left[ \langle E_{DW} \rangle ^2 - \langle E_{DW}^2 \rangle \right] = -\beta \langle \left( \Delta E_{DW} \right) ^2 \rangle \end{aligned}$$

To minimize the Shannon entropy we update $\beta$ using

16 $$\begin{aligned} \delta \beta = -\eta ' \frac{\partial S}{\partial \beta } = \eta ' \beta \langle \left( \Delta E_{DW} \right) ^2 \rangle . \end{aligned}$$

We point out that a different learning rate $\eta '$ was applied relative to the previous RBM learning rate $\eta$ in Eqs. (4)–(7). During the training process, we use $\delta \beta = \eta ' \langle \left( \Delta E_{DW} \right) ^2 \rangle$ for comparisons to linearly ramping $\beta$ and maximizing the likelihood. As shown in Fig. 4, the squared energy terms may increase much faster than the mean energy used in maximizing the likelihood. Therefore, $\eta '$ is set to be $10^{-3}$ of $\eta$. The formation of the QUBO which is the input to the D-Wave hardware is related to $\beta$ as previously given by (9). Therefore $\beta$ scales the energy of the QUBO which can also affect the learning rate of the RBM. In this situation, the value of $\beta$ plays an important role in the Boltzmann distribution that represents D-Wave hardware and RBM training. Limiting the learning rate of $\beta$ avoids very large values so that the QUBO energy in (9) remains larger than energy associated with thermal noise.

### Bayesian statistical analysis

The three techniques for updating the hyperparameter $\beta$ are tested on the D-Wave 2000Q5 machine. See the “Methods” section for details. All methods are evaluated by conducting unsupervised training of $7 \times 7$ pixels of black and white Bars and Stripes (BAS) images. A mini-batch of 500 images was used within a total training set of 1000 images. This results in updating our weights and biases twice per epoch over the entire 1000 image data set. The error of reproducing the images is evaluated on a set of non-trained BAS images (i.e., testing set) using the sum of squares error

17 $$\begin{aligned} Er&= \frac{1}{N_{test}}\sum _{k=1}^{N_{test}} \sum _{i=1}^{49} \left( v_{i k } - v^{rep}_{i k} \right) ^2 \end{aligned}$$

where we define $N_{test}=1000$ as the size of the testing set, $v_{ik}$ and $v_{ik}^{rep}$ represent the white or black pixel values for the testing images and the reproduced images, respectively. The calculations acquire 1000 samples from D-Wave hardware for each epoch and we iterate over 300 epochs in total. This process is repeated 16 times to build statistical estimates when $\beta$ is manually controlled or updated using the maximum likelihood or the minimum Shannon’s entropy approach.

The error at each epoch in (17) is modeled as an exponentially decaying function per epoch to quantify the rate of convergence and the asymptotic error using Bayesian statistics^15^. As seen in Fig. 3, after approximately the first 20 epochs, the decay in error is approximately an exponentially decaying function. Therefore, the following function is used to model the error reduction over epochs

18 $$\begin{aligned} e(i_{epoch}) = Ke^{-\alpha i_{epoch}} + c \end{aligned}$$

where the random parameters ${\overline{\theta }} = [\alpha , K, c]$ define the rate of decay of the error per epoch ($i_{epoch} = [1,\ldots ,N]$). Through Bayesian inference, we identify the posterior distributions for ${\overline{\theta }}$ and how its uncertainty propagates to obtain statistical estimates of the asymptotic error from *c* and rate of convergence from $\alpha$. We also note that we neglect the first 20 epochs where little reduction in error is observed. The random parameters, ${\overline{\theta }}$, are identified using the Markov Chain Monte Carlo (MCMC) method which is numerically implemented using Delayed Rejection Adaptive Metropolis (DRAM)^15,16^. The parameter uncertainty contained in ${\overline{\theta }}$ provides performance metrics for each hyperparameter tuning method.

The error between (17) versus (18) is assumed to be independent and identically distributed (iid) over the epochs which allows us to implement a Gaussian likelihood function in Bayes equation. The prior for all parameters is flat (non-informative) and DRAM is used to minimize the sum of square error according to

19 $$\begin{aligned} e_{model} = \sum _i^{280} \left( Er_i - e_i \right) ^2. \end{aligned}$$

This is equivalent to maximizing the Gaussian likelihood function^15^. The discrepancies between the true error and the modeling error is minimized by sampling the random parameters in ${\overline{\theta }}$. We found that 10,000 samples were sufficient to produce converged posterior distributions based on observations of the parameter chains and choosing multiple initial guesses for ${\overline{\theta }}$.

**Figure 5:**
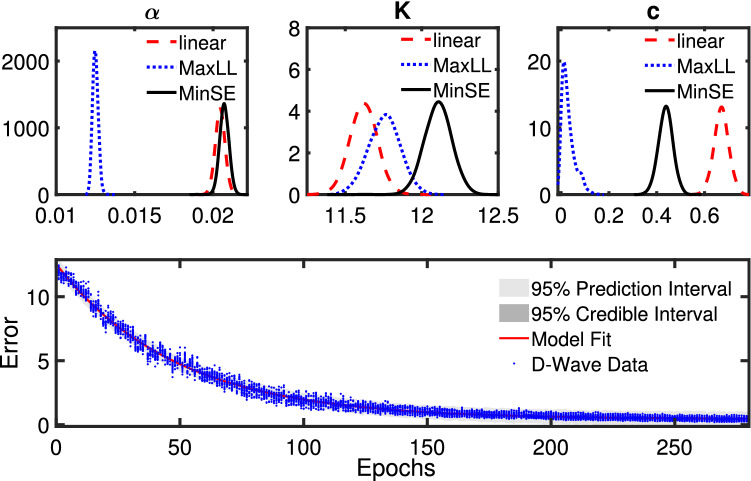
Plots of the Bayesian uncertainty analysis for fitting 16 D-Wave training results to an exponential decay function. All the plots are for $s_{pause}=0.6$. The legends correspond to linear ramping $\beta$ (linear), maximum log likelihood method (MaxLL), and the minimum Shannon entropy method (MinSE).

The Bayesian uncertainty analyses provide quantified uncertainty on convergence rates and asymptotic error estimates contained within posterior densities of $\alpha$ and *c*, respectively. An example of such analysis is illustrated in Fig. 5. The error versus epochs illustrate that an exponential decay model provides a good estimate of the behavior of the D-Wave trained RBM. This plot of image recognition error decay also includes 95% credible and prediction intervals which calculate the uncertainty due to the ${\overline{\theta }}$ parameters and the measurement noise based on the 16 different D-Wave experiments conducted on each algorithm for updating $\beta$. The D-Wave pause time described in the introduction was $s_{pause}=0.6$ for this particular example; see the “Methods” section for additional details on pausing. The three subplots illustrate the Bayesian posterior probabilities for the inverse convergence rate $\alpha$, the initial error based upon *K*, and the asymptotic error from *c*. The results show that the maximum log likelihood method (MaxLL) gives the lowest asymptotic error followed by the minimization of Shannon’s entropy (MinSE), and highest error for the linear ramping of $\beta$ (linear). The maximum likelihood method also has a slightly slower convergence rate as shown in the posterior densities for $\alpha$ while the convergence rate was comparable for minimizing Shannon’s entropy and linearly ramping $\beta$. Recall from Fig. 4, the change in $\beta$ over the epochs for each method shows an increase in $\beta$ is shown to increase over the epochs but at different rates. The maximum likelihood method is the slowest to initially change $\beta$ where its value is 1.5 for about the first 120 epochs and then increases at the fastest rate.

**Figure 6:**
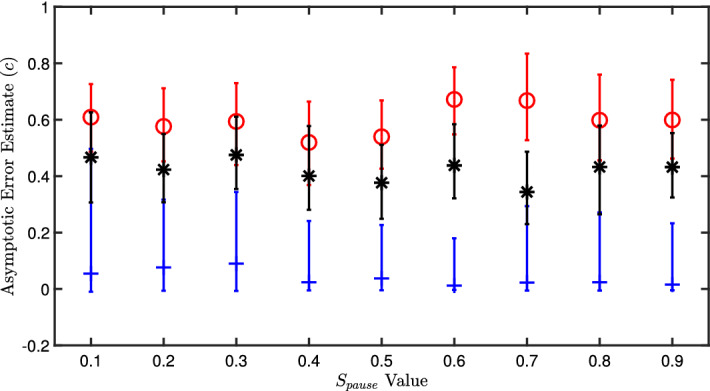
Plot for the three different hyperparameter updating schemes. The red circles present errors of training with linearly ramping $\beta = 1.5 \rightarrow 3$. The blue crosses present errors of training with maximizing the log-likelihood. The black stars present errors of training when minimizing Shannon’s entropy. The marks represent the mode value from each Bayesian posterior of *c*.

Based on Bayesian analysis, we compare the overall performance of the three hyperparameter schemes using the posterior densities for the asymptotic error (*c*). This is done for all different possible pausing values to quantify if the hyperparameter updating is independent of the pausing scheme. Shown in Fig. 6, the mode and 95% probability interval limits of *c* are plotted. These limits were extracted from the posterior densities for *c*, as seen by the example in Fig. 5 for $s_{pause}=0.6$. It is shown that the maximum likelihood method remains superior and independent of the D-Wave pause time. On average, maximizing the log likelihood performs 23 times better than linear ramping $\beta$ and 16 times better than minimizing the Shannon’s entropy. While $s_{pause}=0.8$ and 0.9 are included here, we found that the D-Wave machine automatically pauses the annealing schedule at $s_{pause}=0.8$ therefore there is no difference in these two cases beyond noise within the hardware.

## Discussion

We have developed alternative methods to adaptively update the D-Wave hyperparamter $\beta$ for unsupervised machine learning problems using the Restricted Boltzmann Machine. We find that both maximizing the likelihood and minimizing Shannon’s entropy reduces the steady-state error relative to linearly incrementing $\beta$. In both adaptive schemes, no *a priori* information is needed for $\beta$ which provides a more robust means to train neural networks for different types of data-driven problems. By maximizing the likelihood, $\beta$ is driven by the mean differences in energy of the RBM. In the case of Shannon’s entropy minimization, updates in $\beta$ are driven by the variance of the RBM energy. This is because of the cancellation of average energy as detailed in the “Methods” section. In the image recognition problem, $\beta$ was found to continue to increase unbounded; however, this is limited by finite temperatures on D-Wave hardware. In future work, it would be interesting to include the $\beta ^{-1}$ dependence on $\delta \beta$ in Eq. (12) to assess its effect on RBM error convergence rates and asymptotic error magnitude for a broader range of data-driven problems.

## Methods

### Optimization details

The derivation that leads to Eq. (3) is obtained from the following relations

$$\begin{aligned} \begin{aligned} \frac{\partial ( - \log P ) }{ \partial \theta }&= - \frac{\partial ( \log P ) }{ \partial \theta } = - \left[ \frac{\partial ( \log e^{-E} ) }{ \partial \theta } - \frac{\partial ( \log {\mathbf {Z}} ) }{ \partial \theta } \right] = - \left[ \frac{\partial ( -E ) }{ \partial \theta } - \frac{1}{{\mathbf {Z}}} \frac{\partial {\mathbf {Z}} }{ \partial \theta } \right] = \frac{\partial E }{ \partial \theta } + \frac{1}{{\mathbf {Z}}} \frac{\partial }{ \partial \theta } \left( \sum e^{-E} \right) \\&= \frac{\partial E }{ \partial \theta } + \sum \frac{1}{{\mathbf {Z}}} \frac{\partial }{ \partial \theta } \left( e^{-E} \right) = \frac{\partial E }{ \partial \theta } + \sum \frac{ e^{-E}}{{\mathbf {Z}}} \frac{\partial (-E) }{ \partial \theta } = \frac{\partial E }{ \partial \theta } - \sum P \frac{\partial E }{ \partial \theta } = \frac{\partial E }{ \partial \theta } - \big \langle \frac{\partial E }{ \partial \theta } \big \rangle \end{aligned} \end{aligned}$$

The derivation of (11) which is used in the log-likelihood maximization is

$$\begin{aligned} \frac{\partial ( \log P_{DW} ) }{ \partial \beta }&= - \frac{\partial ( \log P_{DW} ) }{ \partial \beta } = \frac{\partial E_{DW}}{\partial \beta } - \big \langle \frac{\partial E_{DW}}{\partial \beta } \big \rangle = \frac{\partial (E/\beta )}{\partial \beta } - \big \langle \frac{\partial (E/\beta )}{\partial \beta } \big \rangle = -\frac{1}{\beta ^2} ( E - \langle E \rangle ) \end{aligned}$$

Lastly, the derivation of (15) which is used in the minimum entropy method is

$$\begin{aligned} \frac{ \partial S }{ \partial \beta }&= -\sum _i \frac{\partial }{\partial \beta } \left( P_i \log P_i \right) = \underbrace{- \sum _i \left( \frac{\partial P_i }{ \partial \beta } \right) \log P_i}_{\textcircled {1}} + \underbrace{- \sum _i P_i \left( \frac{\partial }{\partial \beta } \log P_i \right) }_{\textcircled {2}} \end{aligned}$$

The first term $\textcircled {1}$ can be explicitly derived as

$$\begin{aligned} {\textcircled {1}}&= - \sum _i \left( \frac{\partial P_i }{ \partial \beta } \right) \log P_i = -\sum _i \left[ \frac{1}{{\mathbf {Z}}} \frac{\partial }{\partial \beta } \left( e^{-\beta E_i} \right) + e^{-\beta E_i} \frac{\partial }{\partial \beta } \left( \frac{1}{{\mathbf {Z}}} \right) \right] \log P_i = -\sum _i \left[ \frac{e^{-\beta E_i}}{{\mathbf {Z}}} \left( -E_i \right) + \frac{e^{-\beta E_i}}{ - {\mathbf {Z}}^2} \frac{\partial {\mathbf {Z}}}{ \partial \beta } \right] \log P_i \\&= -\sum _i \left[ -P_i E_i + \frac{e^{-\beta E_i}}{ - {\mathbf {Z}}^2} \sum _j \frac{\partial }{\partial \beta } \left( e^{-\beta E_j} \right) \right] \log P_i = -\sum _i \left[ -P_i E_i + \frac{e^{-\beta E_i}}{ - {\mathbf {Z}}^2} \sum _j e^{-\beta E_j} \left( -E_j \right) \right] \log P_i \\&= -\sum _i \left[ -P_i E_i + \frac{e^{-\beta E_i}}{ {\mathbf {Z}}} \sum _j \frac{e^{-\beta E_j}}{ {\mathbf {Z}}} \left( E_j \right) \right] \log P_i = -\sum _i \left[ -P_i E_i + P_i \langle E \rangle \right] \log P_i = \sum _i [E_i - \langle E \rangle ] P_i \log P_i \\&= \sum _i [E_i - \langle E \rangle ] P_i \left[ \log e^{-\beta E_i} - \log {\mathbf {Z}} \right] = \sum _i [E_i - \langle E \rangle ] P_i \left[ -\beta E_i - \log {\mathbf {Z}} \right] \\&= \sum _i \beta P_i [E_i \langle E \rangle - E_i^2 ] - \sum _i \left[ E_i - \langle E \rangle \right] P_i \log {\mathbf {Z}} = \beta \left[ \langle E \rangle ^2 - \langle E^2 \rangle \right] - \left[ \langle E \rangle \log {\mathbf {Z}} - \langle E \rangle \log {\mathbf {Z}} \sum _i P_i \right] \\&= \beta \left[ \langle E \rangle ^2 - \langle E^2 \rangle \right] \end{aligned}$$

while the second term $\textcircled {2}$ is zero as seen from

$$\begin{aligned} \textcircled {2}&= -\sum _i P_i \frac{\partial \log P_i}{\partial \beta } = -\sum _i P_i \frac{\partial }{\partial \beta } \left( \log e^{-\beta E_i} - \log {\mathbf {Z}} \right) = -\sum _i P_i \left[ \frac{\partial }{\partial \beta } \left( e^{-\beta E_i} \right) - \frac{\partial }{\partial \beta } \log {\mathbf {Z}} \right] \\&= -\sum _i P_i \left( -E_i \right) - \sum _i P_i \left( - \frac{\partial \log {\mathbf {Z}}}{\partial \beta } \right) = \langle E \rangle - \sum P_i \langle E \rangle = 0. \end{aligned}$$

### Pausing scheme

The D-Wave hardware uses superconducting QPUs to create a quantum Ising spin system in a transverse field. The quantum Ising model has the Hamiltonian^17^

20 $$\begin{aligned} H_{Ising}(s) = - \frac{A(s)}{2} \sum _i {{\hat{\sigma }}}^x_i + \frac{B(s)}{2} \left( \sum _i h_i {{\hat{\sigma }}}^z_i + \sum _{i>j} J_{ij} {{\hat{\sigma }}}^z_i {{\hat{\sigma }}}^z_j \right) \end{aligned}$$

where ${{\hat{\sigma }}}$ are Pauli matrices operating on a qubit. A normalized unitless parameter $s(t) = [0 , 1]$ is introduced as a function of annealing time. At the beginning of annealing, $A(0) \gg B(0)$, which leads to a easily prepared ground state of superposition states. At the end of annealing, $A(1) \ll B(1)$, the eigenstates $\{ \uparrow , \downarrow \}$ of $H_p$, which consists of $\sigma ^z$ only, correspond to spin configurations of the classical Ising model $\{ +1, -1 \}$ given by

21 $$\begin{aligned} E_{Ising} = -\sum _i h_i S_i - \sum _{i>j} J_{ij} S_i S_j \end{aligned}$$

that can be transformed to Eq. (8). The tool suit (Ocean^18^) provides control of annealing in terms of *s*(*t*)^19^. Instead of a linear increase in *s* as a function of time, *s* first increases linearly and then is held constant for a pausing time $t_{pause}$ before increasing linear again until $s(t_{final})=1$.

It has been suggested that controlling the annealing scheme can reduce the effect of noise during the quantum annealing processes^8,9,20^. The pausing scheme^8^ was used in this work to assess its effect on machine learning training and interdependencies on the hyperparameter updating method. No interdependency was identified as shown in Fig. 6.
